# Design and Evaluation of CPR Emergency Equipment for Non-Professionals

**DOI:** 10.3390/s23135948

**Published:** 2023-06-27

**Authors:** Jiayu Xie, Qun Wu

**Affiliations:** College of Art and Design, Zhejiang Sci-Tech University, No. 8 Kangtai Road, Shengtanghe Community, Linping District, Hangzhou 311103, China; 2659965647xiejiayu@gmail.com

**Keywords:** wearable devices, multisensory feedback, intelligent interaction, cardiopulmonary resuscitation, chest compression

## Abstract

Sudden cardiac death is a sudden and highly fatal condition. Implementing high-quality emergency cardiopulmonary resuscitation (CPR) early on is an effective rescue method for this disease. However, the rescue steps of CPR are complicated and difficult to remember, and the quantitative indicators are difficult to control, which leads to a poor quality of CPR emergency actions outside the hospital setting. Therefore, we have developed CPR emergency equipment with a multisensory feedback function, aiming to guide rescuers in performing CPR through visual, auditory, and tactile interaction. This equipment consists of three components: first aid clothing, an audio-visual integrated terminal, and a vital sign detector. These three components are based on a micro-power WiFi-Mesh network, enabling the long-term wireless transmission of the multisensor data. To evaluate the impact of the multisensory feedback CPR emergency equipment on nonprofessionals, we conducted a controlled experiment involving 32 nonmedical subjects. Each subject was assigned to either the experimental group, which used the equipment, or the control group, which did not. The main evaluation criteria were the chest compression (CC) depth, the CC rate, the precise depth of the CC ratio (5–6 cm), and the precise rate of the CC ratio -(100–120 times/min). The results indicated that the average CC depth in the experimental group was 51.5 ± 1.3 mm, which was significantly better than that of the control group (50.2 ± 2.2 mm, *p* = 0.012). Moreover, the average CC rate in the experimental group (110.1 ± 6.2 times/min) was significantly higher than that of the control group (100.4 ± 6.6 times/min) (*p* < 0.001). Compared to the control group (66.37%), the experimental group showed a higher proportion of precise CC depth (82.11%), which is closer to the standard CPR rate of 100%. In addition, the CC ratio of the precise rate was 93.75% in the experimental group, which was significantly better than that of 56.52% in the control group (*p* = 0.024). Following the experiment, the revised System Availability Scale (SUS) was utilized to evaluate the equipment’s usability. The average total SUS score was 78.594, indicating that the equipment’s acceptability range was evaluated as ‘acceptable’, and the overall adjective rating was ‘good’. In conclusion, the multisensory feedback CPR emergency equipment significantly enhances the CC performance (CC depth, CC rate, the precise depth of CC ratio, the precise rate of CC ratio) of nonprofessionals during CPR, and the majority of participants perceive the equipment as being easy to use.

## 1. Introduction

Sudden cardiac death (SCD) [1] is a cardiovascular disease that poses a significant threat to human health. Worldwide, over 3.5 million deaths occur annually due to cardiac arrest [2]. Approximately 80% of these cases take place outside of the hospital environment, referred to as out-of-hospital cardiac arrest (OHCA), which can happen in various settings [3]. The timely implementation of high-quality cardiopulmonary resuscitation (CPR) [4] before the arrival of medical personnel is crucial for the survival of OHCA patients [5,6,7,8]. CPR [9] primarily involves chest compressions (CC) and artificial respiration, and numerous studies have demonstrated that the quality of the CC is a key factor in the success of CPR [10,11]. However, research indicates that the application and success rates of CPR skills in OHCA situations face significant challenges [12]. Reports on the quality of OHCA rescue efforts [13,14] have revealed that the CPR performance of rescuers is generally inadequate. This can be attributed to the complexity of the traditional CPR steps, which are difficult to remember, in addition to the challenge of maintaining quantifiable standards. Even for medical professionals, sustaining high-quality CPR during prolonged resuscitation attempts can be extremely demanding [15]. Moreover, in OHCA cases, the first responders and rescuers are often non-professionals [12,16], and the gap in the knowledge between non-professionals and CPR experts contributes to the suboptimal outcomes in OHCA rescue efforts [17].

There is a significant body of research highlighting the effectiveness of feedback devices in enhancing CPR actions [18,19,20,21]. Early experiments conducted by Kern and colleagues [22] utilized recording devices to guide rescuers in mastering the compression rhythms, demonstrating the positive impact of auditory feedback on CPR actions. However, Kern’s study primarily focused on assessing the CC rate index of CPR actions and did not include CC depth in their assessment indicators. In a subsequent experiment by Tae Nyoung Chung [23], the impact of a metronome guide on CC depth was evaluated, incorporating CC depth as an additional evaluation index alongside Kern’s study. The findings revealed a mutually influential relationship between the CC duty cycle and depth, with an increase in the CC rate leading to an improvement in the CC depth. Kurowski’s study demonstrated that the TrueCPR device significantly enhanced the chest compression effectiveness during simulated CPR processes [24]. Additionally, Buléon’s research indicated that the real-time visual feedback provided by equipment improved the quality of chest compressions and self-efficacy in CPR [25].

Audio-visual feedback (AVF) is a commonly used interactive form of CPR feedback device. AVF feedback devices can be categorized into two types: integrated and independent [26]. Integrated AVF devices are often combined with automated external defibrillators (AEDs) or human models, and they tend to be more expensive, making them suitable for in-hospital emergencies. Independent AVF devices, on the other hand, are designed as separate products that can be worn on the rescuer’s hand or placed on the patient’s chest. For instance, Chiwon Ahn developed a novel chest compression smart ring feedback system [27], which assists rescuers in achieving the ideal depth range during CPR. Yeongtak Song proposed a real-time estimation algorithm for chest compression depth (CCD) based on a smartphone [28]. This concept can be implemented on an Android smartphone to help detect the chest compression depth of rescuers during CPR. Independent AVF devices are typically user-friendly, with low technical complexity and production costs, making them suitable for personal or household use. Iskrzycki’s experiment demonstrated that the use of visual real-time feedback devices significantly improved the quality of CPR [29], while Majer’s study showed that the utilization of TrueCPR devices improved the chest compression parameters in simulated resuscitation scenarios [30].

However, there is currently a lack of excellent CPR feedback devices specifically designed for out-of-hospital cardiac arrest (OHCA) environments on the market. While the studies mentioned above have demonstrated the positive effects of CPR feedback devices in simulated experiments, their effectiveness needs to be reassessed in real OHCA settings due to the greater complexity of actual OHCA conditions [31]. The American Heart Association (AHA) has evaluated CPR smart devices and stated that, so far, no device or product consistently performs well for basic life support (BLS) CPR performed outside the hospital, and no other emergency medical equipment, apart from automated external defibrillators (AEDs), consistently improves the survival rate of OHCA patients. At present, there are two main issues with CPR feedback devices. Firstly, the human–machine interaction is often flawed, and some devices have introduced new challenges for rescuers, such as poor comfort, increased fatigue, and potential risks of injury. In Perkins GD’s study, 95% of the participants reported discomfort in their hands and wrists when using the CPREzyTM device, and one subject even experienced soft tissue damage [32]. Secondly, the devices are not user-friendly, making it difficult for non-professionals to learn the operational methods quickly and accurately, resulting in significant delays in initiating CPR. In Bernhard Zapletal’s study [33], three CPR feedback devices (POCKET CPR, iPhone app PocketCPR, and another device) were experimentally evaluated, and the results indicated the suboptimal overall quality of BLS CPR with all three devices, as well as substantial delays in starting CPR caused by each device.

To tackle the problem of suboptimal CPR quality performed by non-professionals in out-of-hospital cardiac arrest (OHCA) situations, we have developed a portable CPR emergency device with multisensor feedback functions, specifically designed for OHCA environments. This device combines CPR smart technology with wearable devices to provide a comprehensive solution. It utilizes a low-power WiFi-Mesh network to enable the long-term wireless transmission of the multisensor data. The feasibility of the device has been evaluated, demonstrating its potential effectiveness. The primary aim of this study is to address the cognitive differences between the rescue experience of non-professionals and the expert knowledge of CPR by utilizing a low-cost and low-tech approach. The goal is to bridge the gap in knowledge and skills, ensuring that non-professionals can perform high-quality CPR in OHCA scenarios. By integrating multi-sensor feedback functions into a portable and user-friendly device, we aim to enhance the effectiveness and efficiency of CPR performed by non-professionals during OHCA incidents.

## 2. Materials and Methods

### 2.1. System Architecture

**Figure 1:**
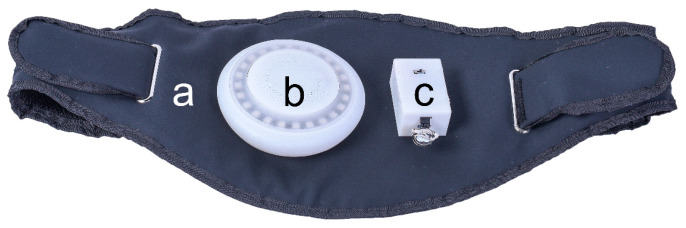
Multisensory feedback CPR Equipment. (**a**) Emergency Clothing, (**b**) Audio-Visual Integrated Terminal, (**c**) Vital Signs Detector.

The system consists of three components, as illustrated in Figure 1: an emergency garment, an audio-visual integrated terminal, and a vital sign detector, each equipped with an ESP32 microcontroller. During operation, the ESP32 microcontrollers in the three components establish a WiFi-Mesh network. The sensors collect the vital signs of the patient and the CPR action data of the rescuer, which are then transmitted via the WiFi-Mesh network to the audio-visual integrated terminal. The system provides feedback to the rescuer through voice prompts and light guidance, enabling them to perform CPR effectively.

**Figure 2:**
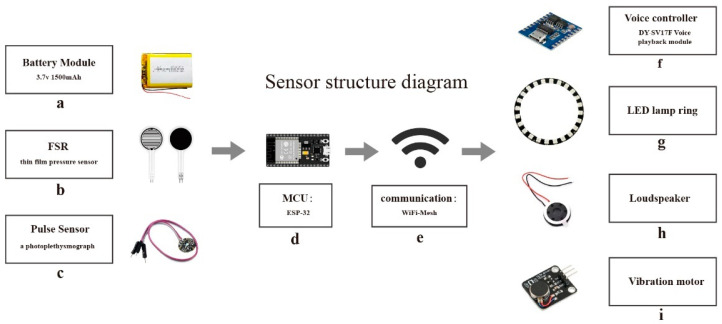
Sensor component diagram of multisensor feedback device (**a**) power supply module, (**b**) FSR thin film pressure sensor, (**c**) pulse sensor of analog heart rate sensor, (**d**) ESP-32 microcontroller, processing data, (**e**) low power consumption Bluetooth, (**f**) DY-SV17F voice broadcast control module, (**g**) LED lamp ring, (**h**) speaker, (**i**) vibration motor module.

This paper focuses on collecting the vital signs of the patient and the CPR compression data of the rescuer using micro-sensors. The CPR action detection and feedback function is achieved through the implementation of WiFi-Mesh network technology [34]. Figure 2 depicts the system architecture, wherein the ESP-32 chip serves as the micro control unit (MCU) of the device. This chip is known for its low power consumption and onboard WiFi function, making it an ideal solution for long-term operation, wireless data transmission, and fulfilling the technical requirements of micro-load applications.

**Figure 3:**
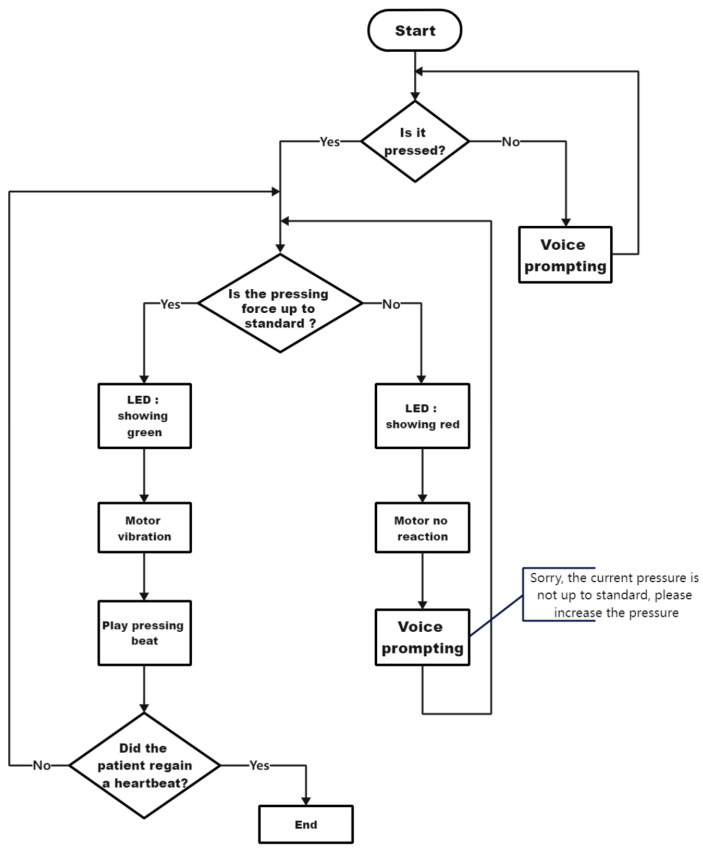
Equipment operation flow chart.

Figure 3 illustrates the operational logic of the device. The vital signs detector collects the patient’s pulse data, which is then used to assess the patient’s physical condition and determine the need for further emergency intervention. The pressure sensor in the first aid clothing detects the compression data from the rescuer. If the quality of the compression does not meet the requirements, feedback is provided to the rescuer.

**Figure 4:**
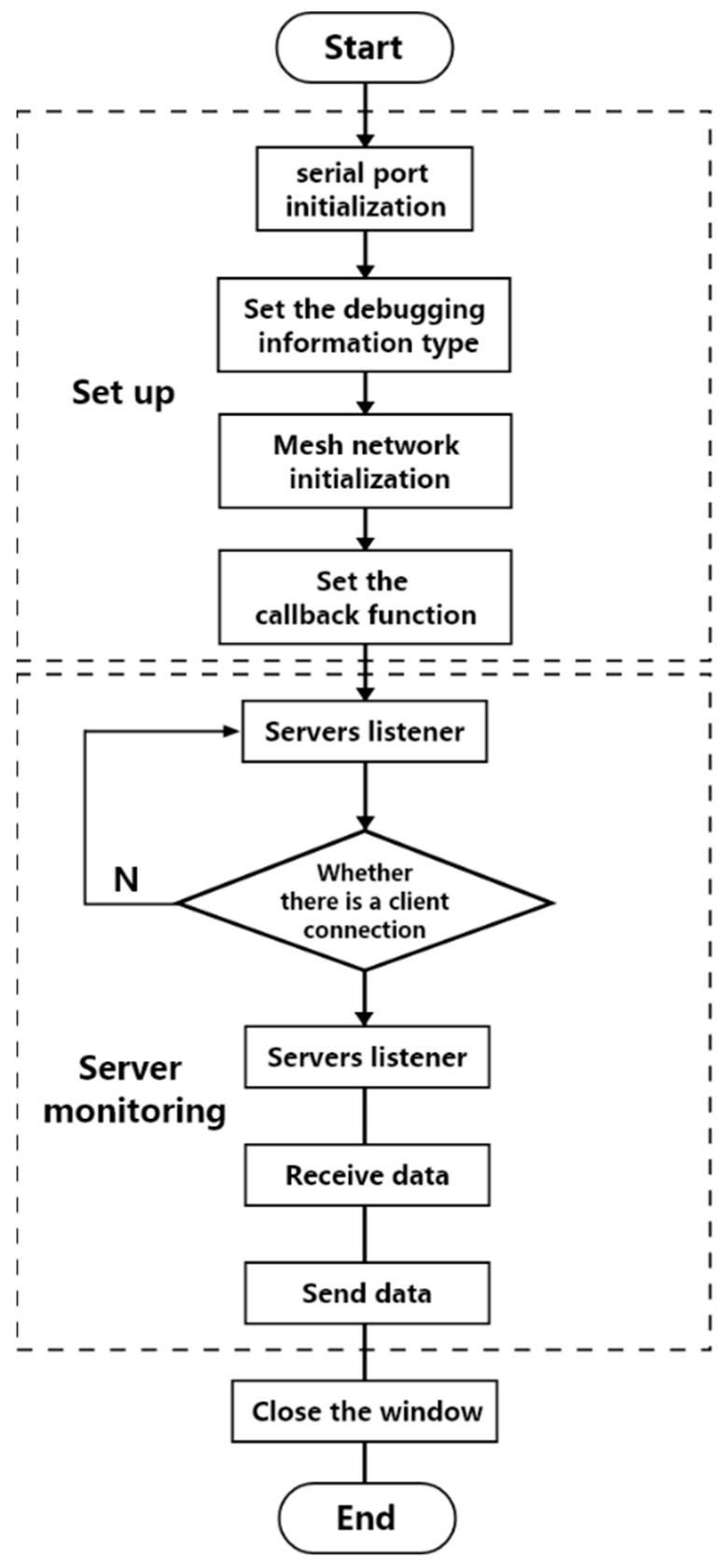
Device control strategy diagram.

The device’s control program is developed using the Arduino platform. Communication is facilitated through the use of ESP-MESH, a network protocol based on WiFi. This protocol enables devices distributed over a large area to connect within the same WLAN. It possesses self-organizing and self-healing capabilities. Figure 4 depicts the control strategy of the device, which consists of two main parts: setup and server monitoring. Upon powering on the device, the program begins with serial port initialization, setting the baud rate to 115,200. Next, the debugging information type and Mesh network initialization are configured. Nodes establish networking by sharing the same network port, account number, and password. The Mesh network callback function is then set to handle ESP-MESH network events. The program’s loop section starts with server monitoring to identify the nodes that meet the connection criteria. Once a connection is established, data exchange occurs with the target node, continuing until the connection is interrupted. The device comprises two clients and a server. The clients are responsible for collecting the pressure and pulse data, respectively. The pulse data is stored as a double-precision floating-point number, while the pressure data is represented as an integer. The client evaluates the data, appends a special byte as an identifier, sends it to the ESP32 on the server, and performs the corresponding operation based on the identifier’s value.

### 2.2. Hardware Design

#### 2.2.1. Emergency Clothing Design

The first aid suit is designed with a structure that is thick in the middle and thin at the ends. It features two slots on each side of the central pressing area, enclosed by metal gas holes. The outer surface of the suit is made of soft polyester, while the internal pressing area utilizes 3 mm thick EVA foam as a buffer material to ensure the comfort of the rescuer during compression.

**Figure 5:**
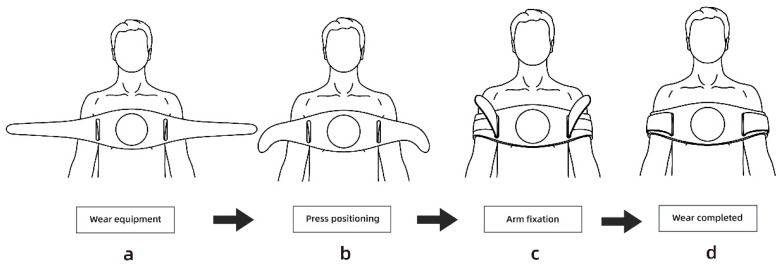
Location of the chest compression area. (**a**) place the device on the chest, (**b**) tie the straps to the patient’s arms, (**c**) pass the straps through the eyelets and secure, (**d**) complete donning.

Figure 5 demonstrates the four-step process of using the emergency clothing. First, the emergency clothes are placed over the patient’s chest. Then, for positioning the pressing area, the strap is threaded through the metal gas eye of the first aid suit and wrapped around the patient’s upper arm. The next step involves correcting the press position and fixing the arm by positioning the upper end of the strap beneath the patient’s armpit and tightly securing the ends of the strap. Finally, the wearing process is completed. It is important to note that the emergency clothing provides a method for positioning the chest compression area for the rescuer. Although patients may have different body shapes, the relative position of the armpit and chest remains consistent. Through testing, it has been determined that the best pressing area for CPR is achieved when the upper arm of the patient is wrapped with the ends of the emergency clothing strap, and the top of the strap is pressed under the patient’s armpit.

**Figure 6:**
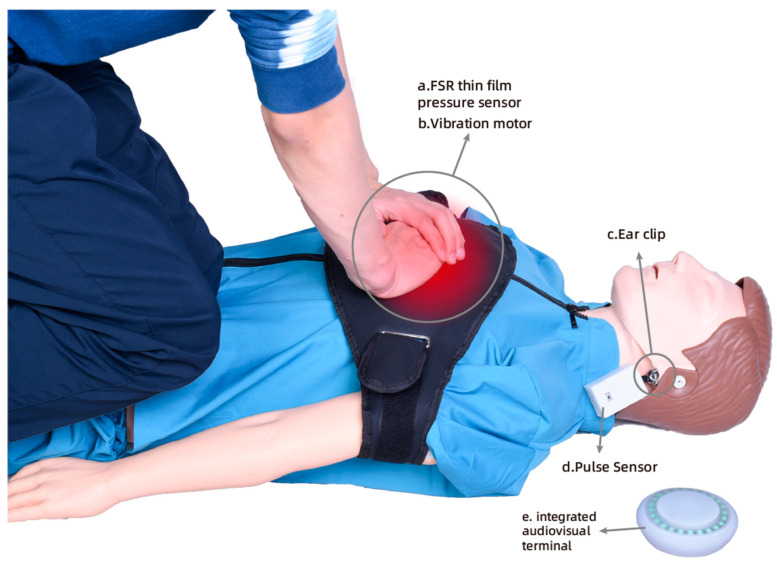
Multisensory feedback diagram.

The hardware circuit of the emergency clothing consists of various components, including sensors, actuators, ESP-32 chips, power modules, and charging modules. The central pressing area of the first aid suit incorporates an FSR thin film pressure sensor, which enables the collection of the pressing data during the rescue process. The thin film pressure sensor has a detection range of 5 kg to 100 kg, equivalent to approximately 49 N–980 N in Newton units, allowing for the detection of pressure data from adults in a kneeling position. Figure 6 illustrates the presence of a built-in micro-vibration motor module within the first aid clothing. When the rescuer achieves the recommended chest compression depth, the vibration motor module activates, providing brief vibration feedback to reinforce the rescuer’s perception of the depth of compression. In the event of abnormal pressing actions by the rescuer, the vibration motor module emits high-frequency vibrations as a reminder to adjust its technique.

#### 2.2.2. Design of Vital Signs Detector

**Figure 7:**
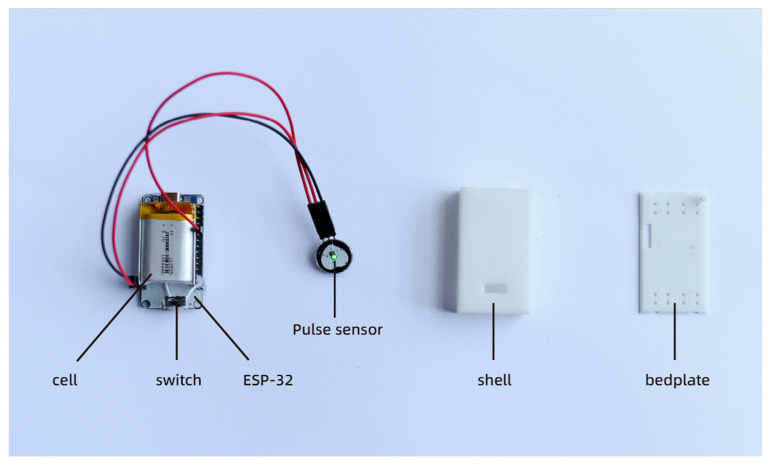
Diagram of the internal structure of the vital signs detector.

In cases of out-of-hospital cardiac arrest (OHCA), it is common for nonprofessional rescuers to make the mistake of discontinuing chest compressions, resulting in a lack of continuous compression. The continuity of the chest compressions is crucial for effective CPR [35]. Stopping compressions before the prescribed time can significantly reduce the quality of rescue efforts. Once it is determined that the patient is experiencing OHCA, immediate CPR must be initiated without an unnecessary assessment of their vital signs, as additional actions can delay the rescue process. To address these challenges, we have developed a vital sign detector using the Pulse sensor, which is an analog heart rate sensor. The Pulse sensor is an open-source hardware device designed for heart rate measurement, pulse waveform measurement, and HRV (heart rate variability) analysis. The sensor operates on the principle of photoelectric volume, where it emits a specific wavelength of light and calculates the pulse frequency based on the changes in light transmittance caused by the beating of the human pulse [36]. In this study, we utilize the Pulse sensor as a simple application without involving its internal development. Figure 7 showcases our vital signs monitor based on the Pulse sensor. It consists of an ESP-32 microcontroller, the Pulse sensor itself, a battery, a switch, a charging module, and a 3D-printed enclosure. The main logic of the program involved in the vital signs detector is as follows: the photodiode in the Pulse Sensor captures the current value corresponding to the reflected light intensity. This current value is then converted to voltage using the circuit and collected by the Analog-to-Digital Converter (ADC) of the single-chip microcomputer, resulting in the pulse waveform curve. The time difference between the peak points of the two adjacent pulse waves is calculated and filtered to obtain the time between the two heartbeats, known as the IBI value. Using the conversion relationship mentioned above, the current heart rate value can be calculated. The program consists of four main parts: sampling, filtering, calculation, and output. During the sampling phase, the pulse analog signal generated by the sensor is primarily collected by the ADC unit at a sampling rate of 500 Hz. The ADC precision is set to 10-bit precision. To address the interference caused by heavy pulse waves resulting from the pulse wave reflection in the artery, the program employs a filtering mechanism. It re-tracks the pulse rise every 0.6 times to mitigate the impact of the heavy pulse waves. In the calculation phase, the heart rate value (BPM) is determined by calculating the difference between the intermediate values of the ascending segments of the two pulse waves, which helps determine the IBI value. The BPM value is then derived using the conversion relationship: BPM = 60/IBI. These four parts, namely sampling, filtering, calculation, and output, form the core components of the program used in the vital signs detector.

The vital signs detector utilizes an ESP32 chip that offers the Light-sleep mode, enabling the device to significantly reduce power consumption to tens of microamperes or even lower. Despite being in this low-power mode, essential functions such as Wi-Fi connection and timers can still be maintained. In Light-sleep mode, the device consumes approximately 100 uA of power. The heart rate sensor, on the other hand, outputs a current of 4 mA, requiring continuous operation. With a battery capacity of 1500 mAh and an output voltage of 3.7 V, the total power consumption of the device amounts to 14.8 mW. Consequently, the equipment can operate continuously for approximately 250 h. It is important to note that this is a theoretical value, and the actual working time may be shorter due to various factors, such as the operating mode, transmission distance, and ambient temperature.

The vital signs detector is used by opening the switch and securing it to the patient’s earlobe or fingertip using the ear clip. It then collects the patient’s pulse signal to detect their vital signs. In typical scenarios, medical professionals use pulse and electrocardiogram data to assess a patient’s heartbeat. However, in OHCA situations, there is often a lack of professional testing equipment, and not all rescuers are trained medical personnel. Therefore, in such cases, pulse signal detection serves as a simple and effective method to evaluate the patient’s physical condition, reducing the need for unnecessary vital signs assessment during CPR.

#### 2.2.3. Audio-Visual Integrated Terminal Design

**Figure 8:**
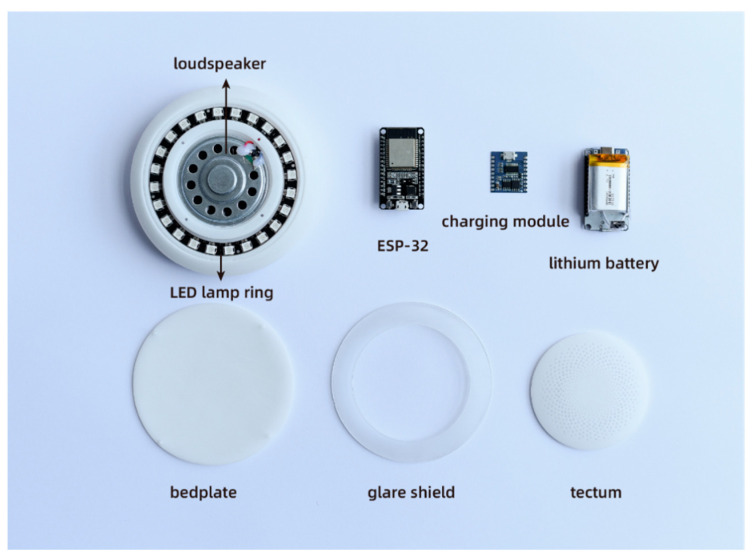
Diagram of the internal structure of the integrated audiovisual terminal.

**Figure 9:**
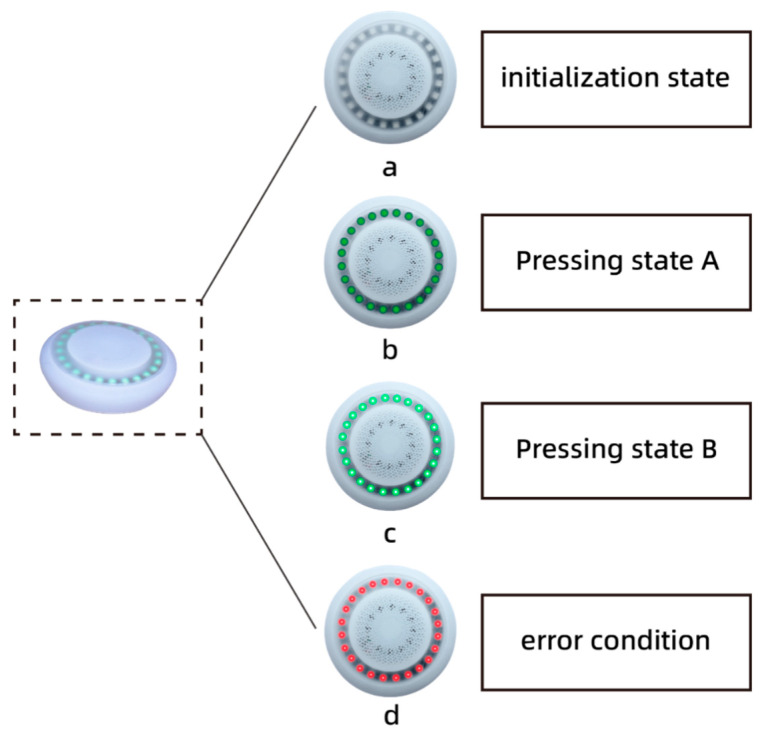
Audiovisual integrated terminal state diagram. (**a**) initial state with no feedback, (**b**) state with weak compression, (**c**) state with normal compression, (**d**) state with abnormal compression.

Figure 8 illustrates the primary hardware circuits of the audiovisual integrated terminal, which consist of the ESP-32 chip, charging module, lithium battery, voice broadcast module, LED lamp ring, and speaker. The ESP-32 chip plays a crucial role in receiving the data from the first aid suits and vital signs detectors. It provides voice broadcasts and light feedback based on the rescuer’s CC depth and CC rate data. The voice broadcast chip offers two modes: beat guidance and voice broadcast. In the beat guidance mode, the device plays a standard pressing beat to guide the rescuer in achieving the correct CC rate. The voice broadcast mode is activated based on specific preset conditions, such as insufficient CC depth, below-standard CC rate, incorrect CC position, or when the patient’s vital signs show signs of recovery. By combining these two modes, rescuers can better grasp the CPR operation method. Figure 9a–c demonstrates the LED lamp ring, which brightens as the CC depth increases. Once the specified depth is reached, the light reaches its maximum brightness. In Figure 9d, when the rescuer’s pressing position is incorrect, a noticeable red light appears, prompting the rescuer to adjust their pressing position or alignment.

### 2.3. Research Program

The purpose of this experiment was to investigate the impact of multisensory feedback devices on the quality of CPR movements performed by non-professionals. A total of 32 subjects were selected and divided into two groups: control group A (CPR without feedback) and experimental group B (CPR with feedback). The quality of CPR actions can be influenced by the physical condition of the subjects. To minimize experimental interference caused by individual differences, a within-subject design was adopted, requiring each subject to participate in both Group A and Group B. Before the experiment, a questionnaire survey was conducted to assess the physical condition of the 32 subjects. All subjects were found to be in normal physical condition, without any muscle injuries, physiological diseases, or other special circumstances. The subjects of this study were nonprofessionals who had not received formal CPR training, and none of the 32 subjects had prior CPR training or rescue experience.

**Figure 10:**
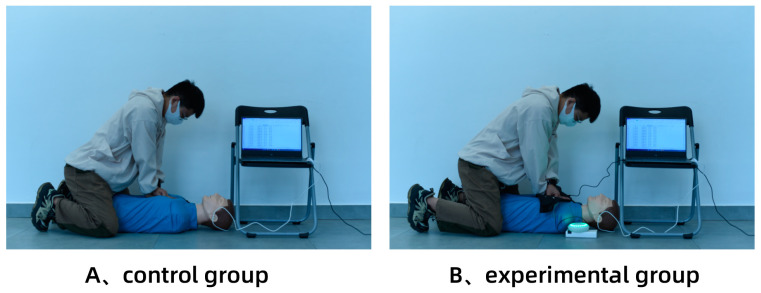
Experimental procedure diagram. (**A**) Control group without equipment, (**B**) Experimental group with equipment.

Before the experiment, a 30 min explanation of CPR actions was provided. After informing the participants about the CPR action standards, a 60 min simulation training session was conducted to ensure that each participant reached a mastery level of CPR skills (subjective assessment). A half-body simulation rubber man was used as an experimental prop and placed on a hard floor. To eliminate the potential interference of the experimental sequence on the results, the 32 subjects were randomly assigned to two groups, with 16 subjects performing the test in order A followed by B, and the remaining 16 subjects performing it in order B followed by A, ensuring that the experimental results were not biased by the experimental sequence. The evaluation indices of this experiment included the depth of chest compressions (CC), the rate of CC, the precise depth of the CC ratio, and the precise rate of the CC ratio. The precise CC depth was defined as 50–60 mm, and the precise rate of the CC is defined as 100–120 times per minute. As depicted in Figure 10, each participant was required to perform a 3 min CPR action test. Considering that CPR requires significant physical energy from the participants, a 1-h break was provided between the two groups to ensure that the participants’ physical strength was fully restored and that fatigue did not impact the experimental results. At the end of the experiment, each participant was asked to complete a questionnaire regarding the CPR emergency equipment with multisensory feedback as the basis for subjective evaluation.

### 2.4. Measurements

In this experiment, the primary focus was on evaluating the CC depth, CC rate, the precise depth of the CC ratio, and the precise rate of the CC ratio of the subjects. The reference standard for the evaluation was the cardiopulmonary resuscitation guidelines introduced by the American Heart Association (AHA) in 2020 [37]. To assess these parameters, a half-body rubber manikin was utilized as the evaluation prop. As the depth of the chest compressions on the half-body rubber manikin is directly related to the applied force, the pressure force exerted by the rescuer was measured and converted into the corresponding CC depth. This measurement was achieved by connecting the rubber manikin to a personal computer via a USB data cable. The computer program received and analyzed the experimental data to calculate the CC depth, the CC rate, the precise depth of the CC ratio, and the precise rate of the CC ratio based on the measured pressure force.

### 2.5. Usability and Availability Evaluation of Devices

The evaluation of the availability of CPR emergency equipment with multisensory feedback was conducted using the revised System Usability Scale (SUS) [38,39]. SUS is a questionnaire designed by Brooke in 1986 for assessing the usability of products or services [40]. It consists of 10 statements, with odd-numbered items representing positive statements and even-numbered items representing negative statements. Two subscales are derived from the scale: the “ease of learning” subscale comprising items 4 and 10, and the “usability” subscale comprising the remaining 8 items. The detailed questions included in the questionnaire are as follows:

1. I will often use this product;
2. I think the product is unnecessarily complex;
3. I think the product is good;
4. I think I need technical support to learn how to use this product;
5. I think the functions of the product are well integrated;
6. I think there are many inconsistencies in the performance of the product;
7. I think most people will learn to use this product soon;
8. I think this product is uncomfortable to use;
9. I have full confidence in using this product;
10. I need to learn a lot before continuing to use the product.

### 2.6. Statistical Analysis

The experimental data were analyzed using SPSS Statistics 26. Descriptive statistics, including the mean ± standard deviation for the continuous variables and the rate/percentage for the categorical data, were employed to summarize the data. The analysis was conducted with a 90% confidence interval (CI). To examine the differences between the subjects before and after using the equipment more thoroughly, a paired sample *t*-test was performed to compare the data between the groups. A significance level of *p* < 0.05 was considered statistically significant, indicating meaningful differences.

### 2.7. Sample Size Determination

Based on a study design for repeated measures, an alpha of 0.05, a power of 90%, and an estimated effect size (primary outcome compression depth, 48.8 ± 5.19 mm versus 53.2 ± 8.8 mm, calculation based on previous data [41]) resulted in a sample size of *n* = 16, at least, for each group.

## 3. Results

### 3.1. Basic Characteristics of Subjects

Table 1 displays the recruitment details of the study, which involved 32 participants, comprising an equal distribution of 16 men and 16 women. Among the participants, 30 had a master’s degree while 2 were undergraduates. The average age of the subjects was 23.72 ± 1.97 (age range: 20–29). Their average height, weight, and BMI were 170 ± 6.21 cm, 60.53 ± 11.36 kg, and 20.82 ± 2.76, respectively. Before the experiment, we ensured that all 32 subjects met the requirements by ruling out any potential physical impairments.

### 3.2. Main Evaluation Results

Table 2 presents the experimental results, demonstrating a significantly better CPR quality in the experimental group compared to the control group. In terms of the CC depth, the average depth in the experimental group was 51.5 ± 1.3 mm, which was significantly superior to the control group’s average of 50.2 ± 2.2 mm (*p* = 0.012, *p* < 0.05), indicating statistical significance. Regarding the CC rate, the average rate in the experimental group was 110.1 ± 6.2 times/min, significantly higher than the control group’s average of 100.4 ± 6.6 times/min (*p* < 0.001). As for the precise depth of the CC ratio, defined as the proportion of CCs with a depth of 5–6 cm to the total CCs, the experimental group (82.11%) demonstrated closer adherence to standard CPR compared to the control group (63.25%), with a *p*-value of 0.066. As for the precise rate of the CC ratio, defined as the ratio of the number of subjects with a mean CC rate of 100–120 beats per minute to the total number of subjects, the experimental group (93.75%) demonstrated closer adherence to standard CPR compared to the control group (56.52%), with a *p*-value of 0.024.

### 3.3. Results of the Equipment Learnability and Usability Evaluation

The SUS scale was assessed on a scale of 100 points, where odd-numbered items were scored on a scale of 1–5 and even-numbered items were scored on a scale of 1–5 (original score). The individual scores for each item were summed and multiplied by 2.5 to calculate the SUS score. A higher SUS score indicates a more favorable rating of the product. As shown in Table 3, the multisensory feedback device received an overall SUS score of 78.594. The average score for usability was 79.88, and the average score for learnability was 73.44. Based on Bangor’s scoring comparison table, the acceptability range was rated as “acceptable” and the overall evaluation as “good”.

## 4. Discussion

Previous research on CPR smart devices has primarily focused on standalone feedback devices, with limited studies exploring the integration of wearable technology with CPR smart devices. This study presents an innovative approach by combining multi-sensory feedback with wearable devices to create a compact and portable CPR emergency device. The device offers guidance and feedback on CPR actions specifically designed for non-professionals. The users of the device demonstrated notable enhancements in the CC depth, CC rate, precise depth of the CC ratio, and precise rate of the CC ratio. Furthermore, all participants successfully utilized and received the feedback from the multi-sensory CPR emergency equipment after a brief explanation, affirming its usability and ease of learning. This experiment revealed significant improvements in several CPR metrics (CC depth, CC rate, precise depth of CC ratio, precise rate of CC ratio) when using the multi-sensory feedback equipment in the experimental group compared to the control group for performing manual CPR. The primary reason for this disparity is that the subjects were non-professionals with limited CPR training time, making the feedback and guidance from the equipment crucial in the CPR process. The fatigue effect among rescuers also contributed to the results, as numerous studies have highlighted its impact on CPR quality [42,43]. Physical exhaustion during CPR was evident, as Hightower observed a decline in the precise depth of the CC ratio from 93% to 39% after 3 min [44]. It is worth noting that rescuers often struggle to perceive a continuous decline in their compression effectiveness [45]. Thus, intelligent interactive devices play a vital role in guiding CPR actions. Although feedback devices cannot provide physical support, they help rescuers assess the correctness of their actions. Even in the face of physical decline, rescuers strive to complete the rescue based on the device’s guidance. The experiment had a duration of three minutes, which could result in a physical decline for many subjects in the later stages of exertion. However, the control group lacked the equipment feedback, making it difficult for subjects to gauge the accuracy of their movements and leading to a more significant decline in CPR quality compared to the experimental group. Furthermore, while the experimental groups demonstrated superiority in the four metrics, the advantage in the CC rate and precise rate of the CC ratio was significantly greater than that in the CC depth and precise depth of the CC ratio. We hypothesize that this disparity in the device’s feedback effect makes rescuers more sensitive to sound feedback than visual and tactile feedback. Two reasons contribute to this observation: firstly, there is a delay in communication between the audiovisual integrated terminal and the first aid clothing, resulting in a mismatch between the rescuer’s compression data and the LED lamp ring’s brightness; secondly, the guidance provided by the vibration feedback for the CC depth is not distinct. Three subjects reported difficulties in discerning changes in the vibration motor module intensity during CPR. Based on the aforementioned results and discussions, we believe that further optimization of the light and vibration feedback is necessary for subsequent studies to provide more significant guidance for rescuers.

The accurate placement of chest compressions is crucial for maintaining high-quality CPR, as incorrect positioning can result in rib fractures 24. Traditional audiovisual feedback (AVF) devices do not facilitate rapid identification of the correct compression position. In conventional CPR protocols, rescuers often have to remove the patient’s clothing to determine the appropriate compression position. This study presents a more efficient and convenient solution. Our findings indicate that CPR movements can be quite intense, making it challenging to secure the traditional equipment onto the patient. To address this issue, we developed a novel type of emergency clothing that serves as the first wearable CPR emergency equipment, leveraging the relative position of the patient’s arms. The emergency clothing is worn by the patient, and secured to the upper arm using straps that pass over the patient’s armpit. This design ensures that the central pressing area of the emergency clothing aligns with the correct compression position.

The CPR emergency equipment with multi-sensory feedback proposed in this study does not fully comply with the requirements of a commercial product. The primary focus of this research was to innovate the interaction mode, and the accuracy of the heart rate data detection was not optimal. However, as the patient’s life status is primarily determined by the presence or absence of a pulse signal, rather than precise pulse information, the sensor’s accuracy has a high fault tolerance rate.

Despite the valuable insights gained from this study, there are several limitations to acknowledge. Firstly, this experiment only considered four assessment criteria, namely the CC depth, CC rate, precise depth of the CC ratio, and precise rate of CC ratio, and did not extensively address other CPR standards, such as the complete chest decompression ratio and flow time. Secondly, the experimental environment was limited to indoor simulations, which may not fully reflect real-world settings outside the hospital, where the equipment would be used. Thirdly, artificial respiration was not included in this study as it focused solely on feedback and guidance for chest compressions. Fourthly, the device does not currently address storage concerns. Therefore, further enhancements are needed before the device can be effectively used in out-of-hospital cardiac arrest (OHCA) scenarios. The CPR multi-sensory feedback device designed in this study does not currently meet the requirements of a commercially available product; rather, it exhibits several limitations. Further efforts are necessary to improve the accuracy of the vital sign detection and assess the power consumption. Nonetheless, this study provides a viable solution to enhance the quality of CPR performed by non-professionals and validates its feasibility. The combination of wearable devices and interactive sensing technology holds promise as a potential new paradigm.

## 5. Conclusions

This study aimed to design and evaluate CPR emergency equipment with multisensory feedback capabilities. The experiments demonstrated the positive impact of the equipment on improving the quality of CPR performed by non-professionals. The development process of the equipment comprised three main components: (1) design and fabrication of emergency clothing; (2) principal research and prototype development of a vital signs monitor; and (3) design and manufacture of an integrated audiovisual terminal.

The evaluation process focused on assessing the effectiveness of the multisensory feedback CPR emergency equipment in guiding the CPR actions of non-professionals. The experimental results indicated that the experimental group utilizing the device exhibited significantly better CPR quality (including CC depth, CC rate, precise depth of CC ratio, and precise rate of CC ratio) compared to the control group performing manual CPR. In the subjective evaluations, the device received an average score of 78.594 on the System Usability Scale (SUS), indicating an “acceptable” level of acceptability and a “good” adjective rating.

This study demonstrates that the combination of intelligent interactive technology and wearable devices effectively bridges the cognitive gap between the rescue experience of non-professionals and the knowledge possessed by CPR experts. Notably, this study presents a novel approach to rapidly position the chest compression site using ergonomic principles. This positioning scheme offers reduced technical complexity and production costs compared to the existing CPR equipment available on the market. It has the potential to be widely deployed as an intelligent CPR device in public spaces. Furthermore, through experimentation, it has been confirmed that the device significantly improves the quality of CPR performed by non-professionals and contributes to mitigating the issue of low CPR skills awareness among the general public to some extent.

## Figures and Tables

**Table 1:** Basic characteristics of experimenters.

Feature	Experimenter (*n* = 32)
Gender (female)	16/32
Age	23.72 ± 1.97
Height (cm)	170 ± 6.21
Weight (kg)	60.53 ± 11.36
BMI (kg/m^2^)	20.82 ± 2.76

**Table 2:** Comparison of the CPR action evaluation between the experimental group and the control group.

Result	A (Control Group)	B (Experimental Group)	Standard CPR	*p*-Value
CC Depth (mm)	50.2 ± 2.2	51.5 ± 1.3	55 ± 7	0.012
CC Rate (times/min)	100.4 ± 6.6	110.1 ± 6.2	113 ± 12	*p* < 0.001
Precise depth of CC ratio (%)	63.25	82.11	100	0.066
**Precise rate of CC ratio** (%)	56.52	93.75	100	0.024

**Table 3:** SUS scale evaluation of multisensory feedback CPR devices.

NO	Question	Mean	SD ^1^
1	I will often use this product	4.16	0.15
2	I think the product is unnecessarily complex	1.72	0.15
3	I think the product is good	4.31	0.122
4	I think I need technical support to learn how to use this product	2.00	0.168
5	I think the functions of the product are well integrated.	4.22	0.125
6	I think there are many inconsistencies in the performance of the product	1.94	0.142
7	I think most people will learn to use this product soon.	4.50	0.110
8	I think this product is uncomfortable to use.	1.66	0.139
9	I have full confidence in using this product	3.69	0.171
10	I need to learn a lot before continuing to use the product.	2.13	0.166
aggregate score		78.594	2.652

^1^ SD = standard deviation.

## Data Availability

Not applicable.

## References

[1] Mehra R. (2007). Global public health problem of sudden cardiac death. J. Electrocardiol..

[2] Mozaffarian D., Benjamin E.J., Go A.S., Arnett D.K., Blaha M.J., Cushman M., Das S.R., de Ferranti S., Després J.P., Writing Group Members (2016). American Heart Association Statistics Committee; Stroke Statistics Subcommittee: Heart Disease and Stroke Statistics—2016 update: A report from the American Heart Association. Circulation.

[3] Berdowski J., Berg R.A., Tijssen J.G.P., Koster R.W. (2010). Global incidences of out-of-hospital cardiac arrest and survival rates: Systematic review of 67 prospective studies. Resuscitation.

[4] Berg K.M., Soar J., Andersen L.W., Böttiger B.W., Cacciola S., Callaway C.W., Couper K., Cronberg T., D’Arrigo S., Deakin C.D. (2020). Adult Advanced Life Support: 2020 International Consensus on Cardiopulmonary Resuscitation and Emergency Cardiovascular Care Science with Treatment Recommendations. Resuscitation.

[5] Abella B.S., Edelson D.P., Kim S., Retzer E., Myklebust H., Barry A.M., O’Hearn N., Hoek T.L.V., Becker L.B. (2007). CPR quality improvement during in-hospital cardiac arrest using a real-time audiovisual feedback system. Resuscitation.

[6] Gallagher E.J., Lombardi G., Gennis P. (1995). Effectiveness of bystander cardiopulmonary resuscitation and survival following out-of-hospital cardiac arrest. JAMA.

[7] Van Hoeyweghen R.J., Bossaert L.L., Mullie A., Calle P., Martens P., Buylaert W.A., Delooz H. (1993). Quality and efficiency of bystander CPR. Belgian Cerebral Resuscitation Study Group. Resuscitation.

[8] Wik L., Steen P.A., Bircher N.G. (1994). Quality of bystander cardiopulmonary resuscitation influences outcome after prehospital cardiac arrest. Resuscitation.

[9] Safar P., Brown T.C., Holtey W.J., Wilder R.J. (1961). Ventilation and circulation with closed-chest cardiac massage in man. J. Amer. Med. Assoc..

[10] SOS-KANTO Study Group (2007). Cardiopulmonary resuscitation by bystanders with chest compression only (SOS-KANTO): An observational study. Lancet.

[11] Rea T.D., Fahrenbruch C., Culley L., Donohoe R.T., Hambly C., Innes J., Bloomingdale M., Subido C., Romines S., Eisenberg M.S. (2010). CPR with chest compression alone or with rescue breathing. N. Engl. J. Med..

[12] Wik L., Kramer-Johansen J., Myklebust H., Sørebø H., Svensson L., Fellows B., Steen P.A. (2005). Quality of cardiopulmonary resuscitation during out-of-hospital cardiac arrest. JAMA.

[13] Stiell I.G., Brown S.P., Christenson J., Cheskes S., Nichol G., Powell J., Bigham B., Morrison L.J., Larsen J., Hess E. (2012). What is the role of chest compression depth during out-of-hospital cardiac arrest resuscitation?. Crit. Care Med..

[14] Aufderheide T.P., Pirrallo R.G., Yannopoulos D., Klein J.P., Von Briesen C., Sparks C.W., Deja K.A., Conrad C.J., Kitscha D.J., Provo T.A. (2005). Incomplete chest wall decompression: A clinical evaluation of CPR performance by EMS personnel and assessment of alternative manual chest compression–decompression techniques. Resuscitation.

[15] Smereka J., Szarpak L., Czekajlo M., Abelson A., Zwolinski P., Plusa T., Dunder D., Dabrowski M., Wiesniewska Z., Robak O. (2019). The TrueCPR device in the process of teaching cardiopulmonary resuscitation: A randomized simulation trial. Medicine.

[16] Abella B.S., Aufderheide T.P., Eigel B., Hickey R.W., Longstreth W.T., Nadkarni V., Nichol G., Sayre M.R., Sommargren C.E., Hazinski M.F. (2008). Reducing barriers for implementation of bystander-initiated cardiopulmonary resuscitation: A scientific statement from the American Heart Association for healthcare providers, policymakers, and community leaders regarding the effectiveness of cardiopulmonary resuscitation. Circulation.

[17] Bobrow B.J., Spaite D.W., Berg R.A., Stolz U., Sanders A.B., Kern K.B., Vadeboncoeur T.F., Clark L.L., Gallagher J.V., Stapczynski J.S. (2010). Chest compression-only CPR by lay rescuers and survival from out-of-hospital cardiac arrest. JAMA.

[18] Grassl K., Leidel B.A., Stegmaier J., Bogner V., Huppertz T., Kanz K.G. (2009). Quality of chest compressions in lay person cardiopulmonary resuscitation. Use of a real-time audiovisual feedback system. Notf. Rett..

[19] Yeung J., Meeks R., Edelson D., Gao F., Soar J., Perkins G.D. (2009). The use of CPR feedback/prompt devices during training and CPR performance: A systematic review. Resuscitation.

[20] Pozner C.N., Almozlino A., Elmer J., Poole S., McNamara D., Barash D. (2011). Cardiopulmonary resuscitation feedback improves the quality of chest compression provided by hospital health care professionals. Am. J. Emerg. Med..

[21] Skorning M.H., Beckers S.K., Brokmann J.C., Rörtgen D.C., Bergrath S., Veiser T., Heussen N., Rossaint R. (2010). New visual feedback device improves performance of chest compressions by professionals in simulated cardiac arrest. Resuscitation.

[22] Kern K.B., Sanders A.B., Raife J., Milander M.M., Otto C.W., Ewy G.A. (1992). A study of chest compression rates during cardiopulmonary resuscitation in humans: The importance of rate-directed chest compressions. Arch. Intern. Med..

[23] Chung T.N., Bae J., Kim E.C., Cho Y.K., You J.S., Choi S.W., Kim O.J. (2013). Induction of a shorter compression phase is correlated with a deeper chest compression during metronome-guided cardiopulmonary resuscitation: A manikin study. Emerg. Med. J..

[24] Kurowski A., Szarpak Ł., Bogdański Ł., Zaśko P., Czyżewski Ł. (2015). The effectiveness of cardiopulmonary resuscitation using CPR feedback devices. Kardiol. Pol..

[25] Lee P.H., Lai H.Y., Hsieh T.C., Wu W.R. (2023). Using real-time device-based visual feedback in CPR recertification programs: A prospective randomised controlled study. Nurse Educ. Today.

[26] Kahsay D.T., Peltonen L.M., Rosio R., Tommila M., Salanterä S. (2023). The effect of standalone audio-visual feedback devices on the quality of chest compressions during laypersons’ CPR training: A Systematic review and meta-analysis. Eur. J. Cardiovasc. Nurs..

[27] Ahn C., Lee S., Lee J., Oh J., Song Y., Kim I.Y., Kang H. (2021). Impact of a Smart-Ring-Based Feedback System on the Quality of Chest Compressions in Adult Cardiac Arrest: A Randomized Preliminary Study. Int. J. Environ. Res. Public Health.

[28] Song Y., Oh J., Chee Y. (2014). A New Chest Compression Depth Feedback Algorithm for High-Quality CPR Based on Smartphone. Telemed. J. E-Health.

[29] Iskrzycki L., Smereka J., Rodriguez-Nunez A., Furelos R.B., Gomez C.A., Kaminska H., Wieczorek W., Szarpak L., Nadolny K., Galazkowski R. (2018). The impact of the use of CPRMeter monitor on the chest compressions quality: A prospective randomized trial, cross-simulation. Kardiol. Pol..

[30] Majer J., Madziala A., Dabrowska A., Dabrowski M. (2018). The place of TrueCPR feedback device in cardiopulmonary resuscitation. Should we use it? A randomized pilot study. Disaster Emerg. Med. J..

[31] Buléon C., Parienti J.J., Morilland-Lecoq E., Halbout L., Cesaréo E., Dubien P.Y., Jardel B., Boyer C., Husson K., Andriamirado F. (2020). Impacts of chest compression cycle length and real-time feedback with a CPRmeter^®^ on chest compression quality in out-of-hospital cardiac arrest: Study protocol for a multicenter randomized controlled factorial plan trial. Trials.

[32] Perkins G.D., Augré C., Rogers H., Allan M., Thickett D.R. (2005). CPREzy: An evaluation during simulated cardiac arrest on a hospital bed. Resuscitation.

[33] Zapletal B., Greif R., Stumpf D., Nierscher F.J., Frantal S., Haugk M., Ruetzler K., Schlimp C., Fischer H. (2013). Comparing three CPR feedback devices and standard BLS in a single rescuer scenario: A randomised simulation study. Resuscitation.

[34] Muhendra R., Rinaldi A., Budimana M., Khairurrijal (2017). Development of WiFi Mesh Infrastructure for Internet of Things applications. Procedia Eng..

[35] Monsieurs K.G., Nolan J.P., Bossaert L.L., Greif R., Maconochie I.K., Nikolaou N.I., Perkins G.D., Soar J., Truhlář A., Wyllie J. (2015). European Resuscitation Council Guidelines for Resuscitation 2015: Section 1. Executive summary. Resuscitation.

[36] Parak J., Korhonen I. Evaluation of wearable consumer heart rate monitors based on photopletysmography. Proceedings of the 2014 36th Annual International Conference of the IEEE Engineering in Medicine and Biology Society.

[37] Panchal A.R., Bartos J.A., Cabañas J.G., Donnino M.W., Drennan I.R., Hirsch K.G., Kudenchuk P.J., Kurz M.C., Lavonas E.J., Morley P.T. (2020). Part 3: Adult basic and advanced life support: 2020 American Heart Association Guidelines for Cardiopulmonary Resuscitation and Emergency Cardiovascular Care. Circulation.

[38] Bangor A., Kortum P.T., Miller J.T. (2008). An empirical evaluation of the system usability scale. Intl. J. Hum. Comput. Interact..

[39] McLellan S., Muddimer A., Peres S.C. (2012). The effect of experience on System Usability Scale ratings. J. Usability Stud..

[40] Brooke J. (1996). Usability Evaluation in Industry.

[41] Semeraro F., Frisoli A., Loconsole C., Bannò F., Tammaro G., Imbriaco G., Marchetti L., Cerchiari E.L. (2012). Motion detection technology as a tool for cardiopulmonary resuscitation (CPR) quality training: A randomised crossover mannequin pilot study. Resuscitation.

[42] Ashton A., McCluskey A., Gwinnutt C.L., Keenan A.M. (2002). Effect of rescuer fatigue on performance of continuous external chest compressions over 3 min. Resuscitation.

[43] Heidenreich J.W., Berg R.A., Higdon T.A., Ewy G.A., Kern K.B., Sanders A.B. (2006). Rescuer fatigue: Standard versus continuous chest-compression cardiopulmonary resuscitation. Acad. Emerg. Med..

[44] Hightower D., Thomas S.H., Stone C.K., Dunn K., March J.A. (1995). Decay in quality of closed-chest compressions over time. Ann. Emerg. Med..

[45] Ochoa F.J., Ramalle-Gomara E., Lisa V., Saralegui I. (1998). The effect of rescuer fatigue on the quality of chest compressions. Resuscitation.

